# Recent trends in research on the role of cholesterol in leukemia: a bibliometric and visualization study

**DOI:** 10.3389/fimmu.2025.1511827

**Published:** 2025-01-23

**Authors:** Huijuan Lv, Ke Lu, Ximing Wang, Yanfang Zhang, Mengqi Zhuang, Jing Li, Keli Su, Wen Gao

**Affiliations:** ^1^ Department of Oncology, Fourth People’s Hospital of Jinan City, Jinan, Shandong, China; ^2^ Department of Lymphoma, Shandong Cancer Hospital and Institute, Shandong First Medical University and Shandong Academy of Medical Sciences, Jinan, Shandong, China; ^3^ Department of Surgery, The Second People’s Hospital of Jinan, Jinan, Shandong, China; ^4^ Department of Laboratory, Fourth People’s Hospital of Jinan City, Jinan, Shandong, China; ^5^ Department of Cardiology, Fourth People’s Hospital of Jinan City, Jinan, Shandong, China

**Keywords:** cholesterol, leukemia, bibliometric analysis, trend, hot spots, Web of Science

## Abstract

**Background:**

Cholesterol metabolism significantly impacts leukemia pathophysiology, affecting tumor cell survival, proliferation, and treatment resistance. This study employs bibliometric analysis and visualization techniques to investigate research trends regarding cholesterol in leukemia and identify key hotspots.

**Methods:**

A systematic search of the Web of Science Core Collection was performed for literature published from 1980 to 2024 using the keywords “cholesterol” and “leukemia,” yielding 1,220 articles. Bibliometric tools like VOSviewer and CiteSpace were utilized for visualizing citation networks and thematic clusters.

**Results:**

The analysis comprised 1,220 publications produced by 6,771 researchers across 1,756 institutions in 68 countries, published in 576 journals with 5,903 unique keywords. Publication output demonstrated a significant rise from 1980 to 2024, peaking in 2022. The United States led in total publications (381) and citations (40,462), followed by China (137 articles) and Japan (102). Notably, U.S. publications had lower average citations than those from Germany and Brazil. Key institutions included the University of São Paulo, Medical College of Wisconsin, and National Cancer Institute, with prominent authors such as Maranhao Raul C. and Girotti Albert W. The journal Cancer Research was the most prolific, while Blood had the highest citation frequency. Major research areas encompassed molecular biology, immunology, and medicine, focusing on the cholesterol-leukemia link. Keyword co-occurrence and co-citation analyses reveal increasing interest in topics like STAT3, multidrug resistance, and treatment interactions. These insights suggest crucial areas for further research.

**Discussion:**

Our findings emphasize cholesterol’s significance in leukemia, indicating its potential as a therapeutic target. Further exploration at the intersection of cholesterol metabolism and leukemia requires multidisciplinary collaboration.

**Conclusion:**

This bibliometric study delineates the evolving research landscape on cholesterol’s role in leukemia, pinpointing emerging trends and future research directions to inform effective therapeutic strategies.

## 1 Introduction

Leukemia, a heterogeneous group of hematologic malignancies characterized by the uncontrolled proliferation of blood cells, is challenging to effectively treat (1). Among the myriad of biological factors that influence the pathophysiology of leukemia, cholesterol metabolism has garnered increasing attention in recent years (2, 3). Cholesterol, a crucial lipid molecule, plays a multifaceted role in cell membrane integrity, numerous signaling pathways, and the regulation of apoptosis (4–6). Given its essential functions, alterations in cholesterol metabolism have potential implications for leukemia initiation, progression, and therapeutic resistance.

The interplay between cholesterol metabolism and cancer biology has become a compelling area of research, revealing that dysregulation of lipid pathways can significantly impact tumor behavior (7, 8). In particular, recent studies have demonstrated that elevated cholesterol levels may contribute to the survival and proliferation of leukemic cells (9) and influence the tumor microenvironment (8). Additionally, elucidating the lipidomic signatures of leukemia could reveal new therapeutic targets and strategies that can enhance treatment efficacy.

With the advent of advanced bibliometric analysis techniques, researchers have the opportunity to quantitatively assess the state of research within specific domains. By evaluating publication trends, citation patterns, and thematic clusters, bibliometric analyses can reveal emerging research hotspots and provide valuable insights into the evolving landscape of a particular field (10). Such insights are crucial for guiding future research directions and fostering interdisciplinary collaborations aimed at addressing complex medical challenges.

In this study, we performed a comprehensive bibliometric analysis to explore the current trends and research hotspots concerning the role of cholesterol in leukemia. By assessing the volume and impact of publications over time and identifying key thematic clusters, we aimed to elucidate the critical intersections between cholesterol metabolism and leukemia research. Our findings not only highlight the progress made in this area but also underscore the potential implications for future investigations and clinical applications.

Ultimately, as research continues to evolve, a nuanced understanding of the role cholesterol plays in leukemia could pave the way for the development of innovative therapeutic approaches that improve patient outcomes in this complex and challenging disease. Through this analysis, we hope to contribute valuable insights that can inform both academic inquiry and clinical practice in the fields of hematology and oncology.

## 2 Methods

### 2.1 Materials and methods

The Web of Science Core Collection (WOSCC) database was used as the primary data source in this study. To ensure the credibility and accuracy of the research results, we used “leukemia” and “cholesterol” as the designated search terms and improved the search method by integrating insights from previous studies (11). The detailed search formulas can be found in the **Supplementary Materials** (**Supplementary Materials**). The publication range spanned 44 years, from January 1, 1980, to July 31, 2024. The articles included in this study were classified as articles or review articles and were written in English. To mitigate potential systematic bias introduced by database updates, we conducted a comprehensive search and screening of publications, which was completed by August 31, 2024.

### 2.2 Data analysis

We created flowcharts using Figdraw (www.figdraw.com) and statistical tables and trend charts using Microsoft Excel 2021. Bibliometric analysis and heatmap generation were performed the bibliometrix 4.1.3 tool in R 4.3.1 software. VOSviewer 1.6.19 and CiteSpace 6.2 R6 were used for the bibliometric analysis of the countries/regions, institutions, authors, journals, keywords, and references of the selected academic studies.

**Figure 1:**
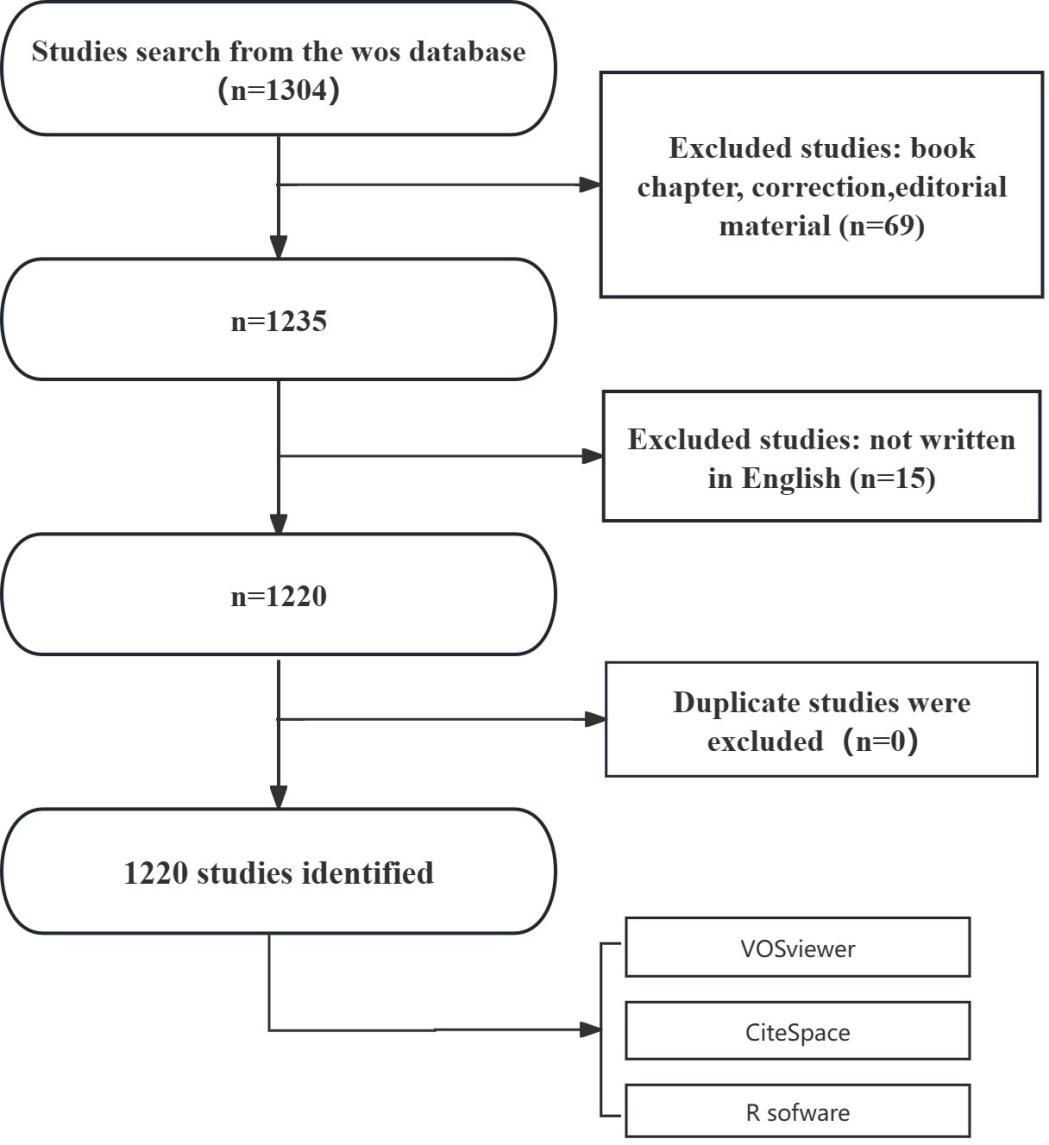
Flowchart of the search strategy and exclusion criteria.

A total of 1,304 published papers were retrieved from the WOSCC database. On the basis of exclusion criteria related to publication date, article type, and language, we ultimately selected 1,220 papers for bibliometric analysis. The specific flowchart is illustrated in **Figure 1**.

## 3 Results

### 3.1 Trends in publication growth

**Figure 2:**
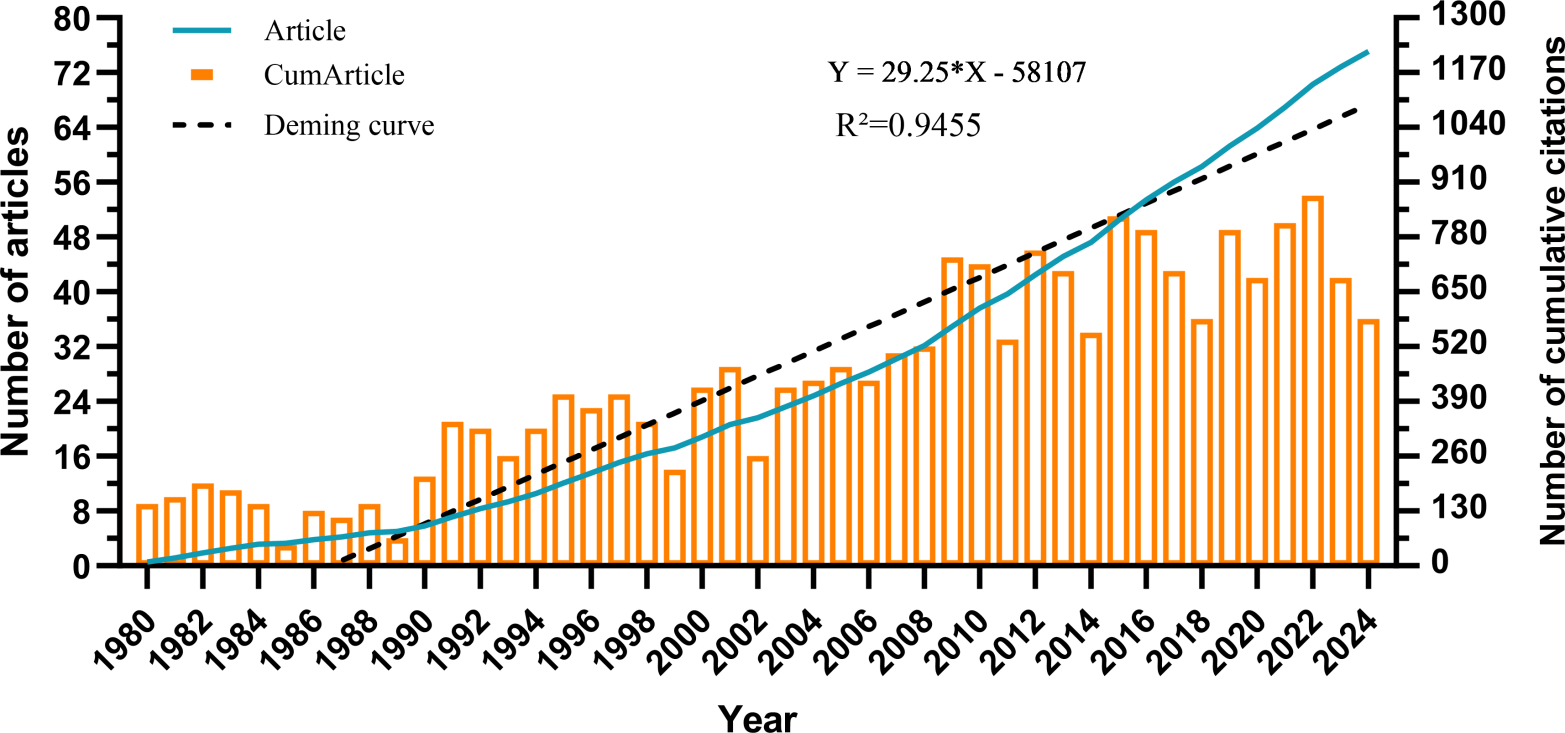
Graph showing the annual and cumulative numbers of publications.

The overall trend in the number of published articles on the role of cholesterol in leukemia from 1980 to 2024 is presented in **Figure 2**. From 1980 to 2024, the number of publications steadily increased each year. The output from 1980 to 1989 was relatively low, averaging fewer than 15 articles per year. However, from 19902006, the number of published articles continuously increased, reaching a cumulative total of 460 articles. Between 2007 and 2024, the number of publications fluctuated, peaking in 2022. This finding indicates that research on cholesterol in the context of leukemia is gradually becoming a popular topic; however, the annual publication volume has not been very stable due to various factors. Nevertheless, the overall trend remains upward.

### 3.2 Statistical analysis of literature on leukemia subtypes

There are a total of 1,220 articles: 414 focused on basic research and 482 on clinical research, alongside 324 review articles. Among the literatures providing information on leukemia subtypes, there are respectively 177 and 161 articles focus on lymphocytic and myeloid leukemia; 267 and 59 papers center on acute and chronic leukemia; 157 and 125 studies concentrate on acute lymphocytic and non-lymphocytic leukemia; and 20 and 41 publications explore the role of cholesterol in chronic lymphocytic and myeloid leukemia.

### 3.3 Countries/Regions and institutions

**Figure 3:**
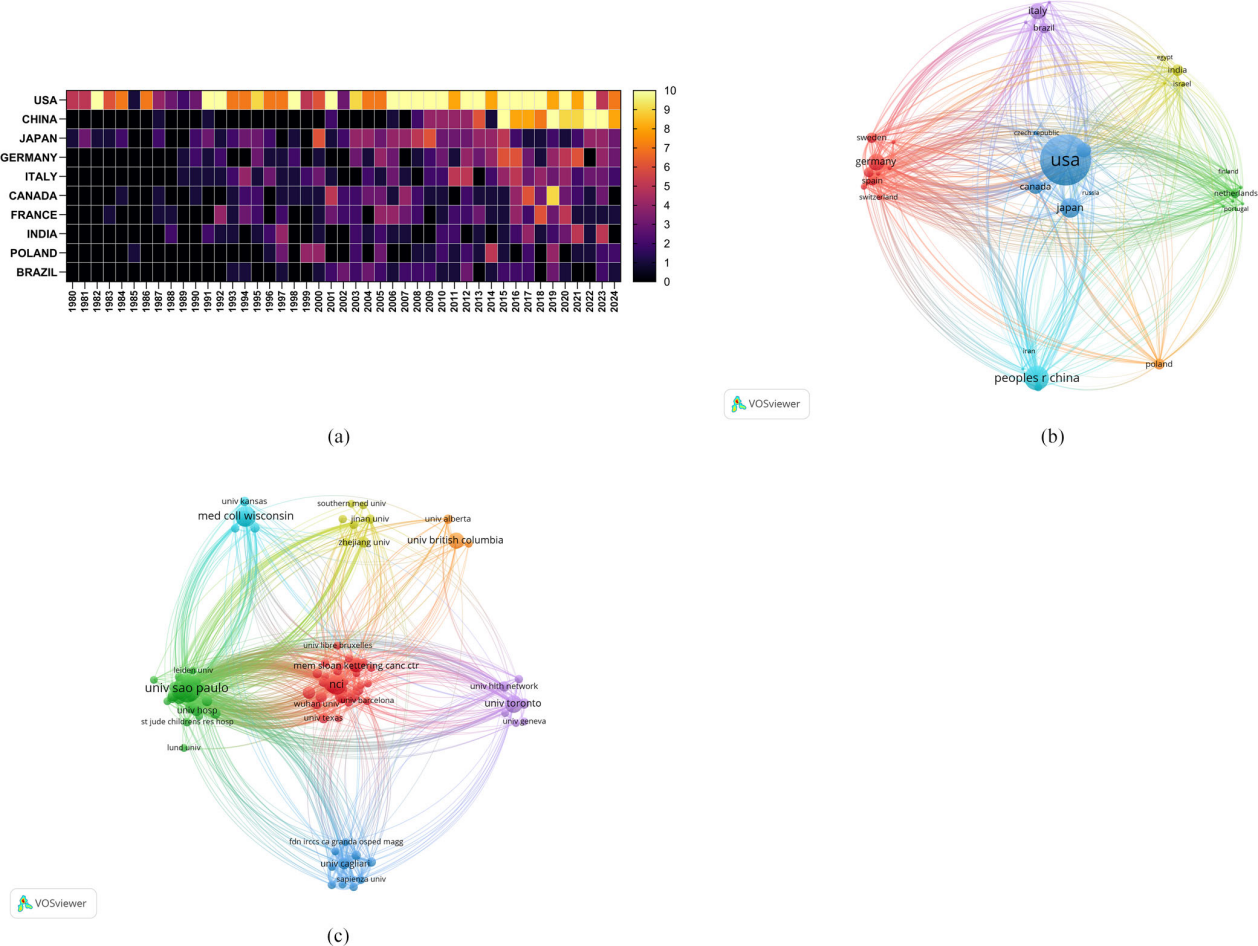
**(A)** Heatmap of the number of annual publications in the top 10 countries/regions included in this study. **(B)** Network map of the countries/regions included in this study. **(C)** Network map of the institutions included in this study.

Researchers from a total of 1,756 institutions from 68 countries/regions have published research on the role of cholesterol in leukemia, as outlined in **Table 1**, which lists the top 10 countries/regions and institutions by productivity. The United States was found to be the primary contributor, with 381 papers, followed by China, with 137 papers, and Japan, with 102 papers. In terms of citation count, the United States leads with 40,462 citations, followed by Germany with 12,584 citations and China with 10,565 citations. Although the United States has the highest number of publications, its average citation count per paper (N=106.20) is significantly lower than that of Germany (N=159.29) and Brazil (N=139.86). Moreover, the average citation counts for China and Japan are relatively low, indicating that authors from high-output countries need to publish higher-quality, more innovative, and widely recognized academic papers in their respective fields. Moreover, the number of annual publications from the United States and China significantly increased in recent years, further demonstrating their leading roles in this domain (**Figure 3A**).

**Table 1:** The top 10 countries/regions and institutions in terms of publications.

Country	Count	Centrality	Citation	Institution	Count	Centrality	Citation
USA	381	0.37	40462	Univ Sao Paulo	37	0.09	1058
CHINA	137	0.05	10565	Medical College of Wisconsin	23	0.02	1887
JAPAN	102	0.14	7474	The NCI	22	0.02	1475
GERMANY	79	0.15	12584	University of Toronto	18	0	1422
ITALY	77	0.04	10451	University of British Columbia	16	0	465
CANADA	71	0.03	8975	Jagiellonian University	15	0.07	435
FRANCE	67	0.14	8368	Northwestern University	14	0.02	1189
INDIA	47	0.06	4306	Cornell University	13	0.08	1438
POLAND	45	0.05	5869	Mem Sloan Kettering Cancer Center	13	0.01	704
BRAZIL	42	0	5874	Karolinska Institute	12	0.03	472

Furthermore, we conducted a network mapping analysis to identify and illustrate the robust communication patterns observed among countries/regions (**Figure 3B**). The size of the nodes indicates the degree of connectivity between countries/regions, with a general centrality greater than 0.1 representing more intense connectivity and greater relevance. The United States had a centrality of 0.37, indicating its role as a bridge in collaboration and paper exchange among various countries. Additionally, Germany (N=0.15), Japan (N=0.14), and France (N=0.14) were also important nodes within this cluster, representing their importance in collaboration among countries/regions.

A total of 1,756 institutions formed 7 major clusters (**Figure 3C**). The University of São Paulo, Medical College of Wisconsin, and the National Cancer Institute (NCI) are the institutions with the highest number of published papers, with 37, 23, and 22 papers, respectively. Furthermore, 5 of the top 10 institutions are located in the United States, indicating a strong interest from American institutions in research on the role of cholesterol in leukemia and highlighting their important position and contributions in this field. The Medical College of Wisconsin has received the most citations (N=1,887), followed by NCIs (N=1,475) and Cornell University (N=1,438), all of which are U.S. institutions. Cornell University has the highest average citation count per paper (N=110.62), followed by Northwestern University (N=84.93) and the Medical College of Wisconsin (N=82.04). Most institutional nodes had centrality values below 0.1, with centrality ranging from 0.010.04, indicating a relatively low level of collaboration among international institutions.

### 3.4 Author and coauthor analysis

**Figure 4:**
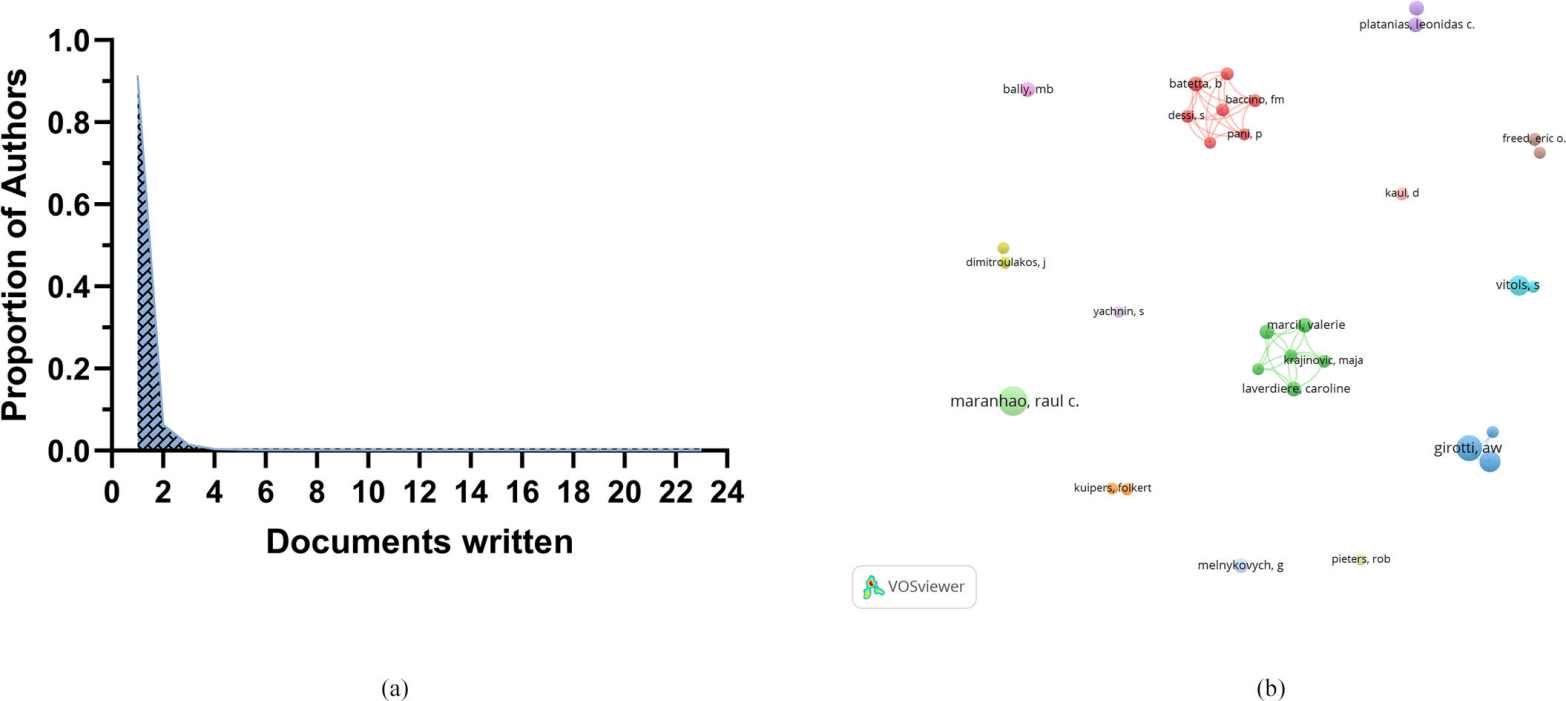
**(A)** Scientific productivity of authors based on Lotka’s law. **(B)** Network diagram of author collaborations.

A total of 6,771 authors participated in research on the role of cholesterol in leukemia. An analysis of scientific productivity using Lotka’s Law revealed that the majority of authors (91.34%) published only one paper (**Figure 4A**). The proportion of those who published two papers is relatively small at 6.31%, whereas only 1.48% have published three papers. Additionally, **Table 2** lists the top 10 authors demonstrating high productivity and citation rates in research on cholesterol in the context of leukemia. Raul C. Maranhao from the University of São Paulo has published the most papers (N=23), followed by Albert W. Girotti from the Medical College of Wisconsin (N=18) and W. Korytowski from Jagiellonian University (N=13).

**Table 2:** The top 10 most prolific and cited authors.

Author	Count	H-Index	Cited-Author	Count	Total Link Strength
Maranhao, Raul C.	23	17	Girotti, A.W.	1782	18
Girotti, Albert W.	18	16	Dimitroulakos, J.	1111	5
Korytowski, W.	13	6	Penn, L.Z	1111	6
Vitols, S.	12	10	Vitols, S.	911	12
Bally, Marcel B.	7	8	Maranhao, Raul C.	879	23
Batetta, Barbara	7	5	Korytowski, W.	490	13
Laverdiere, Caroline	7	6	Freed, Eric O.	402	7
Marcil, Valerie	7	6	Bally, Marcel B.	397	7
Melnykovych, G.	7	6	Platanias, Leonidas C.	360	7
Platanias, Leonidas C.	7	7	Sassano, Antonella	360	7

The authors with the highest citation counts are Girotti, A.W. (N=1,782), Dimitroulakos, J. (N=1,111), and Penn, L.Z. (N=1,111). Moreover, among the top three authors in terms of publication volume, two have relatively high h-index scores: Raul C. Maranhao (N=17) and Albert W. Girotti (N=16). This indicates their important positions and contributions in research on the role of cholesterol in leukemia, establishing them as leading figures in this area of research.

Additionally, other important researchers in the field include S. Vitols from Karolinska University Hospital (12 papers, 911 citations, h-index 10), Marcel B. Bally from BC Cancer Research Institute (7 papers, 397 citations, h-index 8), and Barbara Batetta from the University of Cagliari (7 papers, 252 citations, h-index 5).

Furthermore, our analysis revealed that collaboration among groups of authors, represented by different colors, is quite limited (**Figure 4B**).

#### 3.4.1 Journals and cited academic journals

A total of 576 academic journals actively published research on the role of cholesterol in leukemia. **Table 3** lists the top 10 journals according to the number of publications related to the role of cholesterol in leukemia. The journal with the highest number of published papers is Cancer Research (N=29), followed by Blood (N=22) and Leukemia Research (N=19). Among the top 10 journals, 5 are categorized as Q1 in the Journal Citation Reports (JCR), 2 are categorized as Q2, and 4 have an impact factor greater than 5.

**Table 3:** The top 10 journals and cited journals.

Journal	Count	JCR	IF	Cited Journal	Count	JCR	IF
Cancer Research	29	Q1	12.5	Blood	2140	Q1	21.0
Blood	22	Q1	21.0	Leukemia	1766	Q1	12.8
Leukemia Research	19	Q3	2.1	Journal of Lipid Research	1665	Q1	5.0
PloS ONE	19	Q1	2.9	Cancer Research	1641	Q1	12.5
Journal of Lipid Research	17	Q1	5.0	Physiological Reviews	1156	Q1	29.9
Annals of Hematology	15	Q2	3.0	Annual Review of Pharmacology and Toxicology	1023	Q1	11.2
Journal of Biological Chemistry	15	Q2	4.0	Journal of Biological Chemistry	963	Q2	4.0
Biochemical and Biophysical Research Communications	14	Q3	2.5	Immunity	819	Q1	25.5
Lipids	14	Q3	1.8	Journal of Virology	734	Q2	4.0
Leukemia	13	Q1	12.8	Cancer Chemotherapy and Pharmacology	687	Q2	2.7

In terms of citations, the top 10 cited journals include Blood (N=2,140), followed by Leukemia (N=1,766) and Journal of Lipid Research (N=1,665). Among these, 7 are classified as Q1 and 3 as Q2 in the JCR, with 7 journals having an impact factor greater than 5.

**Figure 5:**
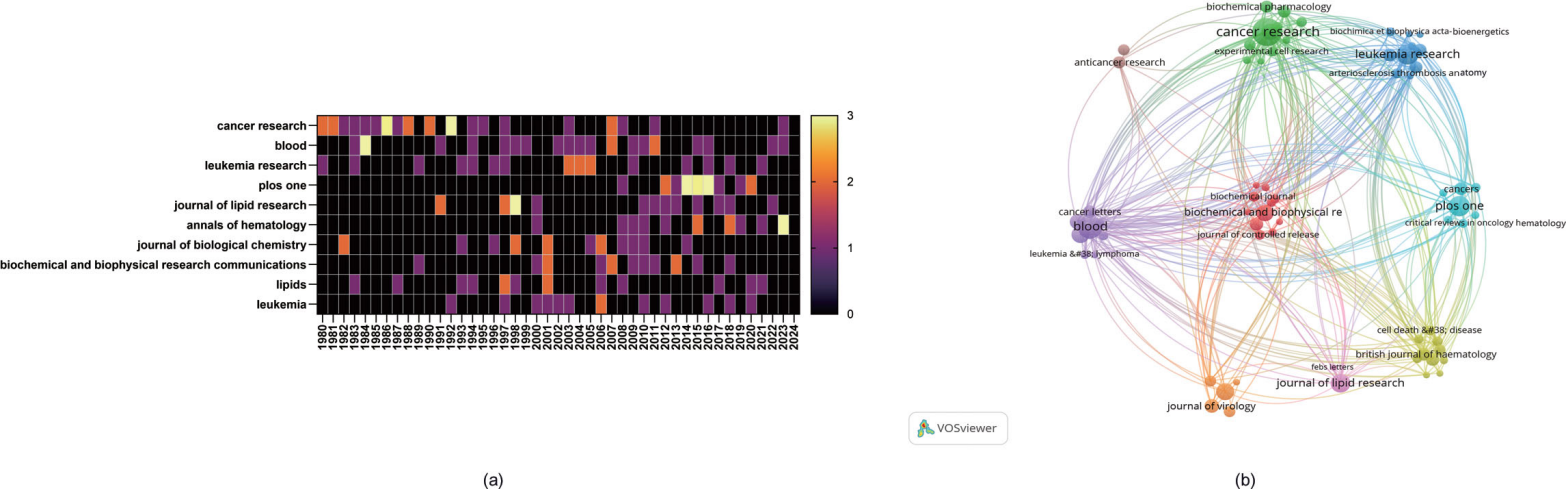
**(A)** Heatmap of annual publications in the top 10 journals. **(B)** Network diagram of author collaborations.

Additionally, there are variations in the publication trends of different journals during the study period. For example, the number of studies published by PLoS ONE significantly increased from 20142016, whereas the number of studies published by Cancer Research had notable spikes in 1985 and 1992. Additionally, the number of studies published by Annals of Hematology significantly increased in 2023 (**Figure 5A**).

The cited journal network map (**Figure 5B**) illustrates the degree of associations between journals. The journal clusters are divided into 9 groups, where the size of the nodes represents the number of cocitations, and the thickness of the edges indicates the strength of the associations. The red cluster, centered on Cancer Research, is the most representative, indicating that these journals have the strongest thematic connections and the highest number of cocitations. Journals of the same color are thematically similar to each other, highlighting their associations with one another regarding research on the role of cholesterol in leukemia research.

### 3.5 Discipline analysis

**Figure 6:**
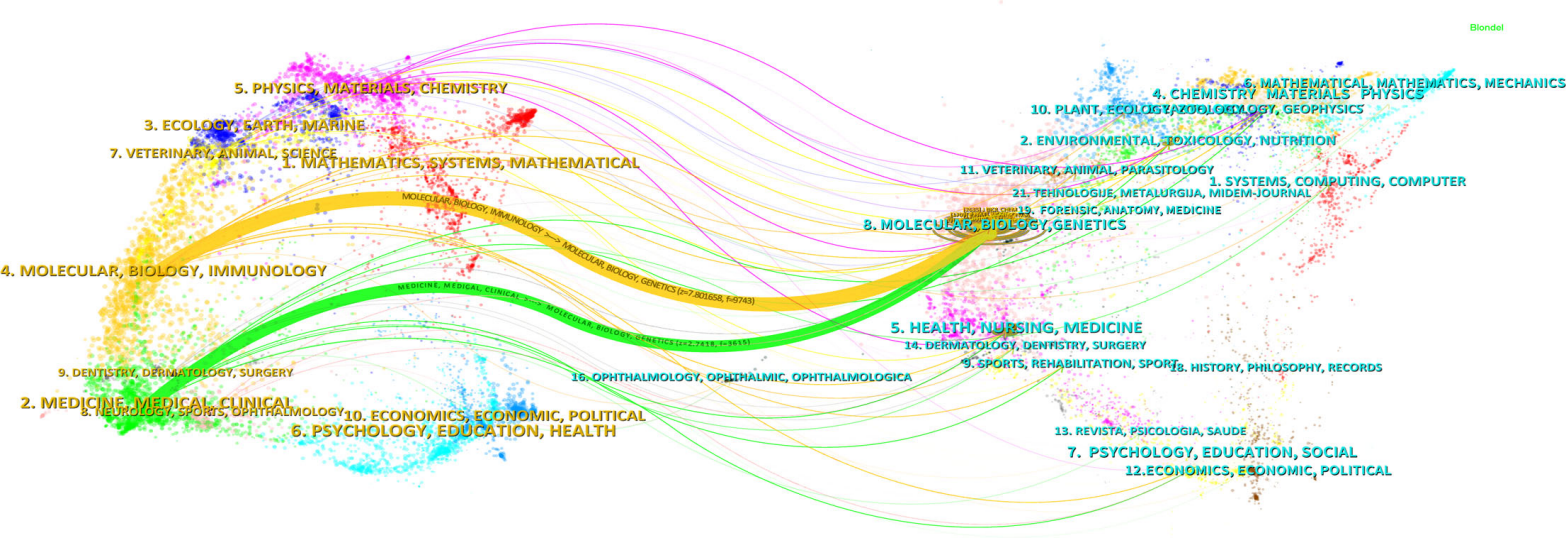
Dual-map overlay of journals included in this study.

The results of the journal dual-map overlay revealed the disciplines involved in the research on cholesterol in the context of leukemia. Research on CAF involves multiple disciplines, as illustrated in **Figure 6**. The visual representation is divided into two parts: the left side shows the citing journals, reflecting the knowledge frontier, whereas the right side displays the cited journals, representing the knowledge base. The lines linking the left and right sections depict citation connections, visualizing the collaborative relationships between the citing and cited journals. These connections reflect the fluidity of primary disciplines within the journals, providing insight into interdisciplinary relationships in the field.

Z-scores emphasize stronger connections, smoother trajectories, and higher scores, represented in the visual representation by the thicker connecting lines. The length of the ellipses indicates the number of authors, whereas the width indicates the number of publications.

As shown in **Figure 6**, the citing journals are primarily distributed in Discipline #2 (Medicine, Health, Clinical) and Discipline #4 (Molecular Biology, Immunology). Conversely, the cited journals are prominently found in Discipline #8 (Molecular Biology, Genetics). Notably, publications in the fields of Molecular Biology and Immunology (yellow trajectory) are influenced by Molecular Biology and Genetics (z=7.80, f=9,743). Additionally, publications in Medicine, Health, and Clinical (green trajectory) are significantly impacted by publications in Molecular Biology and Genetics (z=2.74, f=3,615).

### 3.6 Co-citation, clustering, timeline, and citation bursts

**Table 4** lists the top 10 most cited papers on the role of cholesterol in leukemia, with the study by Aranda, A. et al. titled “Nuclear Hormone Receptors and Gene Expression” being the most cited (N=1,156). This article discusses the nuclear hormone receptor superfamily, which includes receptors for thyroid and steroid hormones as well as orphan receptors. These receptors can be regulated by lipid metabolism products such as fatty acids, prostaglandins, and cholesterol derivatives, influencing gene expression through their interactions with DNA. As ligand-inducible transcription factors, they can induce transcriptional repression in the absence of ligands or activate transcription upon ligand binding. This highlights the role of cholesterol in regulating transcriptional activity and points to potential research avenues for intervening in gene transcription.

**Table 4:** The top 10 most cited articles.

Title	Doi	Journal	Author	Citations
Nuclear hormone receptors and gene expression	*10.1152/physrev.2001.81.3.1269*	*PHYSIOLOGICAL REVIEWS*	Aranda, A	1156
Serum-cholesterol level and mortality findings for men screened in the multiple risk factor intervention trial	*10.1001/archinte.152.7.1490*	ARCHIVES OF INTERNAL MEDICINE	NEATON, JD	596
HMG-coa reductase inhibitors and the malignant cell: the statin family of drugs as triggers of tumor-specific apoptosis	*10.1038/sj.leu.2402476*	LEUKEMIA	Wong, WWL	501
Nanomedicine review: clinical developments in liposomal applications	*10.1186/s12645-019-0055-y*	CANCER NANOTECHNOLOGY	Beltrán-Gracia, E	306
Temperature-, concentration- and cholesterol-dependent translocation of L- and D-octa-arginine across the plasma and nuclear membrane of CD34+ leukaemia cells	*10.1042/BJ20061808*	*BIOCHEMICAL JOURNAL*	Fretz, MM	211
Pharmacokinetic, pharmacodynamic, and pharmacogenetic determinants of osteonecrosis in children with acute lymphoblastic leukemia	*10.1182/blood-2010-10-311969*	*BLOOD*	Kawedia, JD	196
Increased sensitivity of acute myeloid leukemias to lovastatin-induced apoptosis: A potential therapeutic approach	*10.1182/blood.V93.4.1308.404k08_1308_1318*	*BLOOD*	Dimitroulakos, J	192
Hypocholesterolemia in malignancy due to elevated low-density-lipoprotein-receptor activity in tumor-cells - evidence from studies in patients with leukemia	*-*	*LANCET*	VITOLS, S	192
Cholesterol-modulating agents kill acute myeloid leukemia cells and sensitize them to therapeutics by blocking adaptive cholesterol responses	*10.1182/blood-2002-07-2283*	*BLOOD*	Li, HY	175
RP5-833A20.1/mir-382-5p/NFIA-Dependent Signal Transduction Pathway Contributes to the Regulation of Cholesterol Homeostasis and Inflammatory Reaction	*10.1161/ATVBAHA.114.304296*	*ARTERIOSCLEROSIS THROMBOSIS AND VASCULAR BIOLOGY*	Hu, YW	158

**Figure 7:**
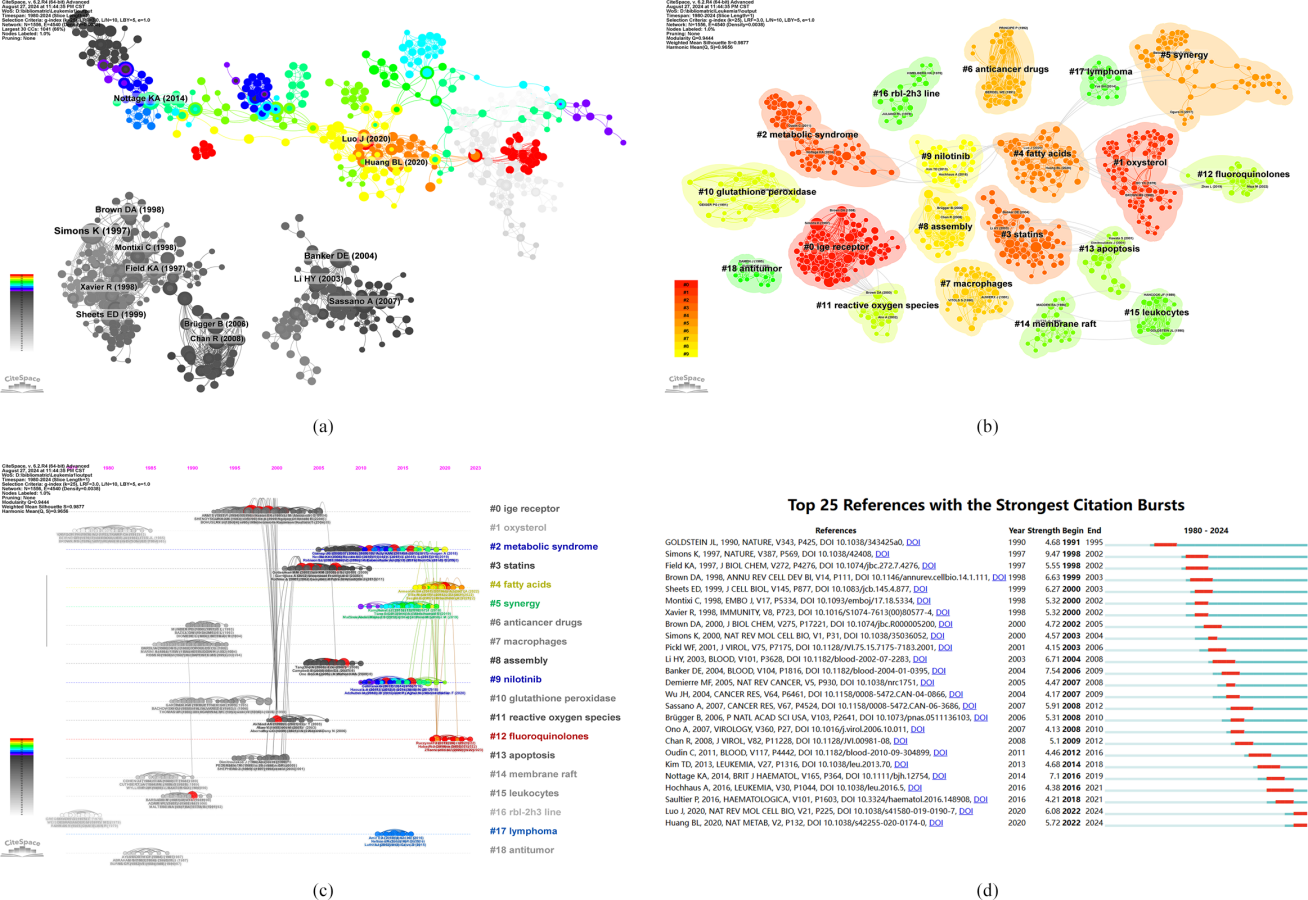
**(A)** Visualization of literature cocitations. **(B)** Literature cocitation clustering. **(C)** Publishing timeline of the literature. **(D)** The top 25 references with the strongest citation burst intensities.

A cocitation analysis of the literature revealed the progress in research on the role of cholesterol in leukemia, as depicted in **Figure 7A**, demonstrating 14 significant nodes. Notably, the majority of widely cited articles emerged between 1990 and 2010. These observations highlight the rapid development and substantial achievements in this field within a specific time period.

**Figure 7B** illustrates the clustering of cocited literature on the role of cholesterol in leukemia, revealing thematic classifications within this area. The diagram displays 19 clusters, with the first named “#0 IgE receptor,” the second “#1 oxysterol,” and the third “#2 metabolic syndrome.” These clusters correspond to hot topics in various stages of cholesterol research in the context of leukemia.

**Figure 7C** presents a timeline view of the clusters formed by cocited literature. Nodes aligned along the same line represent a single cluster, each identified by a label on the right. The larger the node is, the higher the cocitation frequency is, indicating that the nodes that appear on the left generally correspond to classic or relatively outdated topics, whereas those on the right correspond to more recent emerging themes. Several new research hotspots include “#2 metabolic syndrome,” “#4 fatty acids,” “#5 synergy,” “#9 nilotinib,” “#12 fluoroquinolones,” and “#17 lymphoma.”

### 3.7 Keywords co-occurrence, clustering, and outburst

The analysis of cocitation bursts helps elucidate the duration of research on popular topics. To visually represent this, **Figure 7D** was created, where the green line corresponds to the period from 1980 to 2024, and the red line indicates the duration of the citation bursts. Notably, Deborah E. Banker’s 2004 article, “Cholesterol synthesis and import contribute to protective cholesterol increases in acute myeloid leukemia cells” (2006-2009, intensity 7.54), presented the highest citation burst intensity. This study revealed that cholesterol synthesis inhibitors may increase the efficacy of standard leukemia therapies, potentially necessitating tailored treatment strategies for individual leukemia patients. Additionally, Kerri A. Nottage’s article, “Metabolic syndrome and cardiovascular risk among long-term survivors of acute lymphoblastic leukemia - From the St. Jude Lifetime Cohort” (2016-2019, intensity 7.1), posits that adult survivors of childhood acute lymphoblastic leukemia (ALL) face an elevated risk of cardiovascular disease, which is closely linked to dyslipidemia, obesity, and hypertension.

From the 1,220 papers, a total of 5,903 keywords were extracted. **Table 5** presents the top 20 high-frequency keywords based on their count rank. After removing noninformative keywords, the most popular keywords identified were “metabolism” (121 occurrences), followed by “apoptosis” (105), “lipid rafts” (71), “atherosclerosis” (62), and “low-density lipoprotein” (60).

**Table 5:** High-frequency keywords.

Rank	Keyword	Counts	Rank	Keyword	Counts
1	metabolism	121	11	liposomes	45
2	apoptosis	105	12	differentiation	40
3	lipid rafts	71	13	chemotherapy	39
4	atherosclerosis	62	14	inflammation	39
5	low-density-lipoprotein	60	15	pathway	39
6	children	55	16	long-term survivors	38
7	plasma-membrane	54	17	statins	38
8	receptor	53	18	breast-cancer	36
9	inhibition	50	19	gene-expression	36
10	therapy	46	20	protein	36

**Figure 8:**
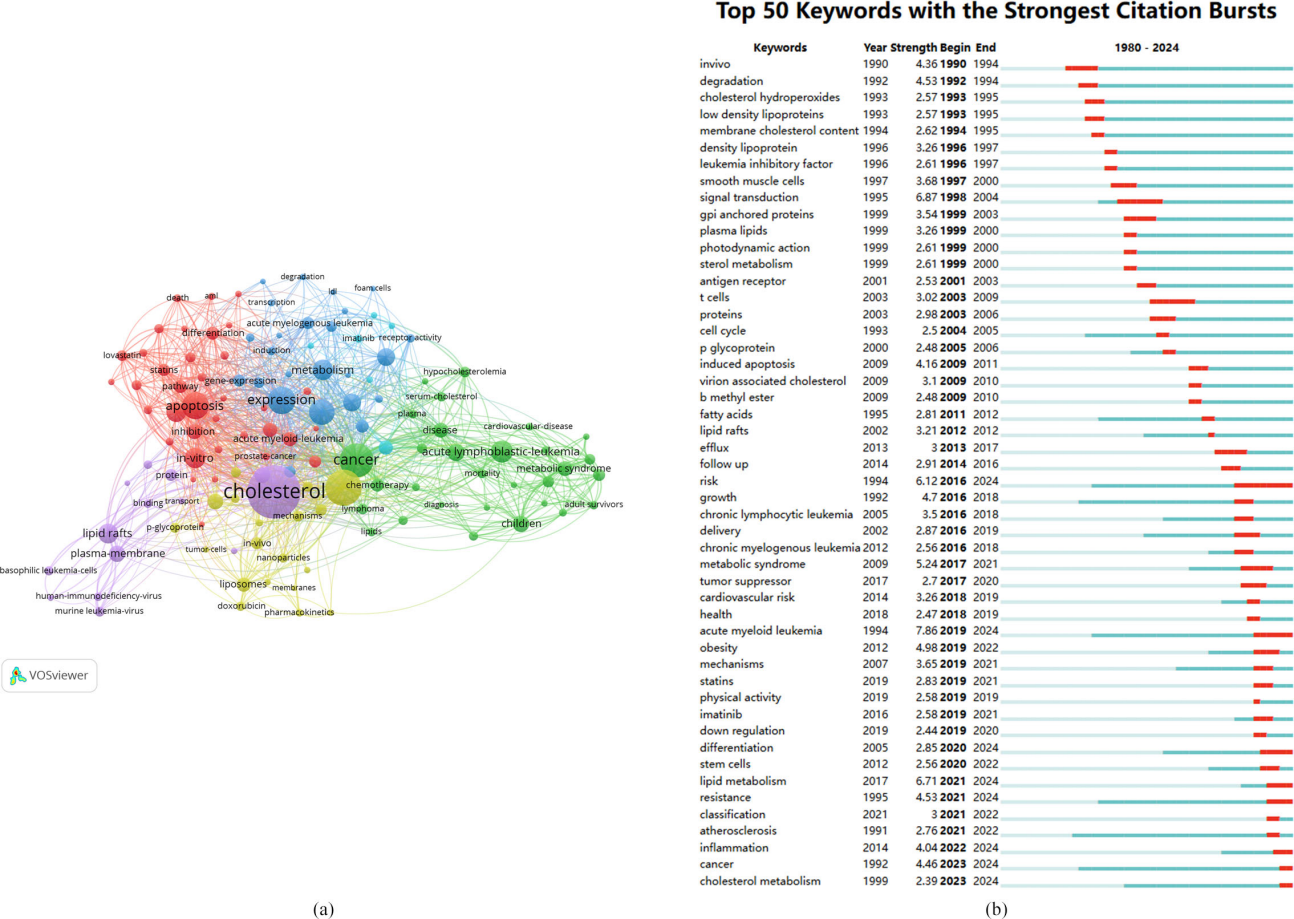
**(A)** Network map of keywords. **(B)** The top 50 keywords with the strongest citation burst intensities.

The analysis of keyword clustering offers insights into the thematic distribution of cholesterol research in the context of leukemia, enhancing the clarity of specific research topics within this field. **Figure 8A** presents a visual representation of the keyword network, revealing the presence of six distinct clusters.

The main red cluster primarily encompasses studies related to the mechanisms of leukemia development, including keywords such as “apoptosis,” “death,” “cholesterol metabolism,” “proliferation,” and “NF-kappa-B.”

The green cluster focuses on the relationship between cholesterol and the onset of leukemia, incorporating keywords such as “macrophage,” “receptor activity,” “atherosclerosis,” “inflammation,” “messenger RNA,” and “low-density lipoprotein.”

The blue cluster mainly investigates various risk factors and diagnostic studies related to cholesterol in leukemia, featuring keywords such as “cardiovascular disease,” “obesity,” “diagnosis,” “bone marrow transplantation,” “mortality,” “chemotherapy,” “children,” and “long-term survivors.”

The yellow cluster primarily addresses the establishment of leukemia research models, including terms such as “murine leukemia virus,” “GPI-anchored protein,” “basophilic leukemia cells,” “model,” “receptor,” and “membrane cholesterol.”

The purple cluster focuses on novel prevention and treatment strategies for leukemia based on cholesterol, with keywords such as “nanoparticle,” “liposomes,” “lipid peroxidation,” “cytotoxicity,” “oxidative stress,” “resistance,” and “delivery.”

The light blue cluster centers on the treatment of acute leukemia, featuring keywords such as “imatinib,” “inhibitor,” “lymphoma,” “nilotinib,” and “therapy.”

This clustering of keywords helps elucidate the multifaceted research landscape concerning the role of cholesterol in leukemia, highlighting areas of active investigation and emerging topics.

The analysis of keyword citation bursts provides insights into the popularity and use of keywords over time, as illustrated in **Figure 8B**.

In the early period (1980-2000), the keywords with citation bursts included “cholesterol hydroperoxides,” “low-density lipoproteins,” and “leukemia inhibitory factor.”

In the middle period (2001-2019), there was a significant increase in the occurrence of keyword citation bursts, particularly in studies related to leukemia pathogenesis. Notably, the keywords used during this period included “antigen receptor,” “cell cycle,” “induced apoptosis,” “tumor suppressor,” and “lipid rafts.”

From 2020 to 2024, the primary focus in cholesterol research related to leukemia shifted toward mechanisms of leukemia, highlighting key areas such as “stem cells,” “lipid metabolism,” “inflammation,” “resistance,” and “classification atherosclerosis.”

This temporal analysis underscores the evolving landscape of research on the role of cholesterol in leukemia, reflecting changes in focus and emerging areas of interest over time.

## 4 Discussion

**Figure 9:**
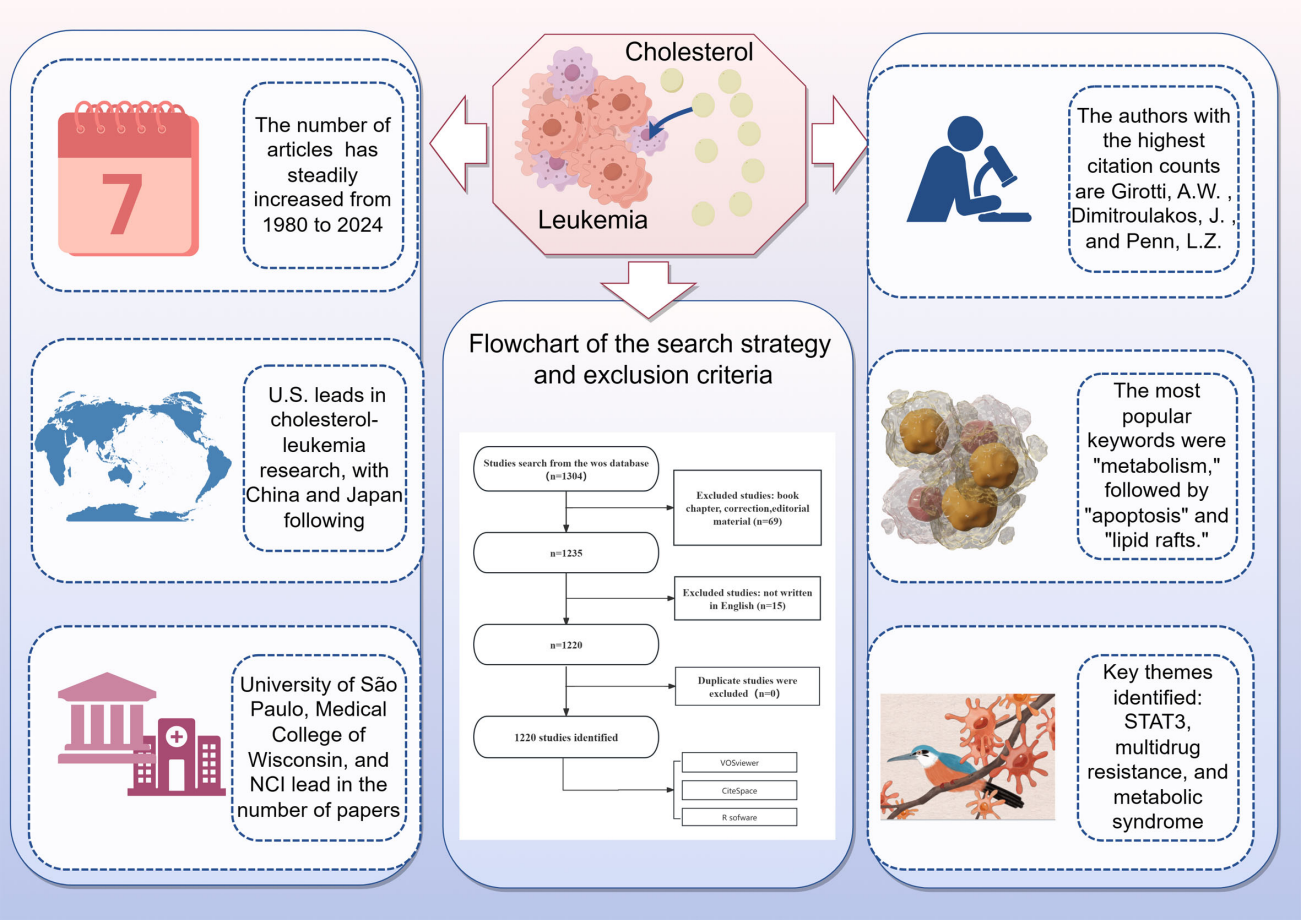
Cholesterol's Role in Leukemia: Recent Trends and Visualization. Cholesterol metabolism significantly affects leukemia by influencing tumor cell survival, growth, and treatment resistance.1,304 studies were identified from the WoS database. After excluding book chapters, corrections, and editorial material (n=69), 1,235 studies remained. Studies not written in English (n=15) were further excluded, leaving 1,220 studies. The number of articles has steadily increased from 1980 to 2024. The United States leads in cholesterol-leukemia research, followed by China and Japan. The University of São Paulo, Medical College of Wisconsin, and NCI are among the institutions with the highest number of publications. The authors with the highest citation counts are Girotti, A.W., Dimitroulakos, J., and Penn, L.Z. The most popular keywords were "metabolism," followed by "apoptosis" and "lipid rafts." Key themes identified include STAT3, multidrug resistance, and metabolic syndrome.

The multifaceted role of cholesterol in leukemia has garnered increasing attention from researchers over recent decades (12). Given the role of cholesterol metabolism in leukemia, we conducted a bibliometric analysis using publication data from the Web of Science (WOS). Our bibliometric analysis of 1,220 selected papers illuminated significant trends in publication growth and highlighted contributions from various countries and institutions. Our objective was to elucidate the current trends and prominent research directions regarding the role of cholesterol in leukemia. On the basis of the consistent upward trend observed in global publications, the role of cholesterol in leukemia has garnered great interest among researchers, with 54 relevant articles published in 2022, the highest number to date. Additionally, as of now, 36 relevant publications have been published in 2024, indicating that the role of cholesterol in leukemia remains a popular research topic (**Figure 9**).

The observed steady increase in the number of publications regarding the role of cholesterol in leukemia from 1980 to 2024 underscores its rising prominence as a popular research area. While the publication output was relatively low from 1980 to 1989, a sustained increase from 19902006 indicates that foundational research laid the groundwork for more extensive inquiries. The peak in publications in 2022 signals a growing interest among researchers, thought to be fueled by advancements in understanding the relationships between lipid metabolism and cancer biology.

This upward trend, albeit punctuated by fluctuations in annual publication volume between 2007 and 2024, reflects the complexity of the field and the influence of factors such as emerging collaborations, funding availability, and advances in research technologies. The overall robustness of publications highlights the increasing recognition of the critical implications of cholesterol in leukemia pathogenesis, treatment response, and immune modulation.

The geographical analysis of the research contributions highlighted some notable disparities. The United States has the highest number of publications (381) and citations (40,462), establishing itself as a nucleus of research activity in the cholesterol-leukemia domain. China’s great publication output (137 papers) indicates a strong commitment to research in this area, although relatively low average citation counts were observed. This disparity implies that while there may be a high volume of research being conducted, there is a need for enhanced focus on the quality and innovation of academic papers.

The status of the United States as a primary contributor can be attributed to its well-funded research infrastructure and collaborative academic culture. Moreover, the scientific infrastructure in China illustrates a common challenge faced by rapidly advancing research landscapes, where higher publishing outputs may sometimes correlate with lower quality of research produced. Promoting international collaboration could increase the academic impact of the work produced in these regions, facilitating innovative exchanges of ideas and leading to the publication of high-quality research.

The network mapping analysis demonstrated a robust communication pattern among countries, with the United States having the highest centrality score (0.37). This suggests that it plays a crucial role in the collaboration among global institutions. Countries such as Germany, Japan, and France also play crucial roles in global scientific collaboration, which reveals a concerted effort among developed nations to address the role of cholesterol in leukemia.

However, the overall centrality values for many institutions were low, reflecting relatively limited cross-institutional collaboration, particularly among developing nations. To harness the potential of international research, strategies that foster collaborative networks should be prioritized. Interdisciplinary collaboration, particularly among oncologists, lipid specialists, and immunologists, can foster innovation in research on the complex interactions between cholesterol and leukemia.

Our analysis revealed that a substantial number of authors contributed to cholesterol-leukemia research, with the majority (91.34%) publishing only one paper. This indicates a challenge to sustain long-term research engagement among authors, which can be important in ensuring continuous progress in scientific understanding. Although leading authors such as Maranhao, Girotti, and Korytowski have made impactful contributions, this landscape reflects a need for deeper, sustained research initiatives by a broader pool of scientists.

Limited collaboration among authors presents both challenges and opportunities. While the presence of leading researchers is critical, the lack of extensive cooperative efforts may restrict the sharing of expertise and resources. To enhance interdisciplinary research, systems that incentivize collaborative projects and foster mentorship among researchers are crucial.

Additionally, the bibliometric analysis identified “Cancer Research” as the journal with the most publications in the field, followed by “Blood” and “Leukemia Research.” The presence of these reputable journals in high-citation categories exemplifies their importance as platforms for disseminating vital research findings on the role of cholesterol in leukemia. Importantly, 5 of the top 10 journals are classified as Q1 according to the Journal Citation Reports, indicating their relevance and authority in the academic community.

The high citation counts achieved by specific journals signal not only the quality of the publications but also the engagement of the authors with top-tier journals. Researchers aspire to publish in these high-impact journals to ensure that their findings reach a wide audience and influence clinical practices. Additionally, the utilization of nanomedicine and advanced therapeutic approaches in the context of leukemia suggests emerging intersections where novel industries are anticipated to thrive.

Our analysis offers valuable insights into the multidisciplinary nature of research focused on the role of cholesterol in leukemia. The evident concentration of studies within the fields of medicine, molecular biology, and immunology reflects how intertwined these fields are. As demonstrated by the dual- map overlay, publications originating from disciplines such as molecular biology have a direct impact on immunology research and clinical applications.

Bibliometric analysis of keyword clustering and citation bursts provides insights into research hotspots, prevailing trends, and their duration of popularity over time. The results of this study indicate that the keywords associated with citation bursts in 1980-2000 primarily included cholesterol hydroperoxides, low-density lipoproteins, and leukemia inhibitory factor (LIF). Studies have shown that elevated levels of cholesterol hydroperoxides are linked to leukemic cell proliferation and survival (13). They promote the activation of downstream signaling pathways, such as the NF-κB (14)and JAK/STAT (15)pathways, which are implicated in cell survival and proliferation. Mechanistically, oxidized LDL (oxLDL) can stimulate inflammatory responses and contribute to a protumorigenic microenvironment (16). This process has been linked to the development of drug resistance in leukemia, highlighting the importance of targeting lipid metabolism to overcome treatment failure (17). Moreover, LIF activates the JAK/STAT pathway in leukemic cells, promoting their growth and resistance to apoptosis (18, 19). Elevated levels of LIF in the bone marrow microenvironment can create a supportive niche for leukemic stem cells, thus contributing to disease relapse. Recent studies suggest that LIF may influence lipid metabolism and the accumulation of cholesterol derivatives, including hydroperoxides (20, 21). Elucidating the relationship between LIF signaling and cholesterol homeostasis could lead to the identification of new therapeutic targets. The inhibition of LIF signaling has shown promise in preclinical models of leukemia. The combined targeting of LIF and oxidative cholesterol metabolism may synergistically enhance treatment efficacy and improve patient outcomes.

The number of keyword citation bursts significantly increased in 2001-2019, particularly in studies related to the leukemia pathogenesis, and the keywords included antigen receptor, cell cycle, induced apoptosis, tumor suppressor, and lipid rafts. Recent studies have revealed that aberrant signaling through antigen receptors can lead to uncontrolled proliferation in leukemic cells. For example, constitutive activation of B-cell receptor (BCR) signaling in B-cell leukemia promotes survival and growth, making it a potential target for therapy (22). Proteins such as cyclins, cyclin-dependent kinases (CDKs), and their inhibitors play critical roles in modulating the cell cycle (23). In leukemia, the overexpression of certain cyclins has been linked to disease progression and poor prognosis. The effectiveness of CDK inhibitors has been investigated in recent studies, and these inhibitors can induce cell cycle arrest and subsequently apoptosis in leukemic cells (24). Combining these inhibitors with conventional therapies may increase treatment efficacy.

In studies on blastic plasmacytoid dendritic cell neoplasms, liver X receptor (LXR) agonists were shown to restore cholesterol efflux and trigger cell apoptosis (14). Investigation of apoptotic pathways revealed that several proapoptotic signals, such as those mediated by p53 (25) and mitochondrial factors (26), are often impaired in leukemic cells. Targeting these pathways could restore apoptotic sensitivity. Researchers, including Pierre-Luc Mouchel, have discovered that the cholesterol metabolite dendritic acid A (DDA) synergizes with anthracycline drugs, exhibiting potent antileukemic activity in acute myeloid leukemia (AML) cells through the induction of lethal autophagy (27).

Lipid rafts are microdomains within the plasma membrane that play pivotal roles in cellular signaling (28). Recent studies have revealed their role in leukemia, particularly in B-cell lymphoma. Lipid rafts aggregate numerous signaling molecules, including those involved in antigen receptor signaling. In leukemic cells, these rafts can facilitate aberrant signaling that drives tumor cell proliferation and survival (22, 29–31).

From 2020 to 2024, research on cholesterol in the context of leukemia has focused primarily on the mechanisms underlying the disease. Key areas of focus included stem cells, lipid metabolism, inflammation, resistance, and the classification of atherosclerosis. Stem cells, particularly leukemic stem cells (LSCs), are central to the pathogenesis and therapy resistance of leukemia (32, 33). LSCs exhibit unique properties, including self-renewal and differentiation capabilities, which contribute to disease progression and relapse (34, 35). Recent studies indicate that these stem cells are often resistant to standard therapies, making them critical targets for intervention (36). Alterations in lipid metabolism have been increasingly implicated in leukemia, affecting cellular functions, signaling pathways, and treatment responses (37, 38). Abnormal lipid metabolism, characterized by increased fatty acid synthesis and altered cholesterol metabolism (39, 40), has been associated with increased proliferation and survival of leukemic cells. For example, leukemic cells often exhibit an upregulation of fatty acid synthase (FASN), promoting a more aggressive phenotype (41). Therapeutic strategies targeting lipid metabolism are being explored. Inhibitors of key enzymes involved in lipid biosynthesis may sensitize leukemic cells to conventional therapies. Ongoing research aims to clarify how specific lipid fractions influence leukemic cell behavior and treatment resistance (40, 42, 43).

Moreover, chronic inflammation in the leukemic tumor microenvironment enhances resistance to therapy and promotes disease progression (44, 45).

Inflammatory cytokines: Cytokines such as IL-6 (46, 47), TNF-α (48, 49), and others contribute to a proinflammatory environment that supports leukemic cell survival. High levels of these inflammatory markers are correlated with poor outcomes in patients (50, 51).

The classification of atherosclerosis, a condition characterized by chronic inflammation and lipid accumulation (52–54), is now being examined in relation to leukemia (55–57). Recent studies suggest that mechanisms driving atherosclerosis, such as dysregulated lipid metabolism (58, 59) and inflammatory responses (60), are similar to those involved in leukemia. The inflammatory aspects of the leukemic microenvironment may parallel those of atherosclerotic lesions, further complicating disease management.

Clinical relevance: Investigating the complex relationships between inflammation, cholesterol metabolism and leukemia can lead to new innovative classification systems for leukemia that incorporate inflammatory and metabolic factors, helping clinicians better predict disease activity and response to treatment.

Future efforts to investigate the role of cholesterol in leukemia through multidisciplinary methods will shed light on how cholesterol metabolism can be manipulated for therapeutic gains. For example, exploring how cholesterol influences immune cell differentiation and functionality in the tumor microenvironment provides insights into the development of new immunomodulatory strategies for leukemia management.

## 5 Conclusion

The role of cholesterol in leukemia is a burgeoning area of research that provides a critical basis for understanding tumor biology and developing innovative therapeutic approaches. Our bibliometric analysis revealed significant trends in publication growth, geographic disparities, influential authors, and thematic directions in the field. The extensive body of research on cholesterol’s role in leukemia, including 1,220 publications from 1980 to 2024, highlights its critical involvement in tumor cell survival, proliferation, and treatment resistance, with particular focus on areas like STAT3 signaling and multidrug resistance. While this substantial evidence base supports considering cholesterol as a promising therapeutic target, further experimental and clinical validation is necessary to confirm the efficacy of cholesterol-related interventions in enhancing leukemia treatments. By fostering greater collaboration, enhancing the quality of research, and prioritizing interdisciplinary studies, we can harness the potential of cholesterol-related research to improve clinical outcomes for leukemia patients.

## Data Availability

The original contributions presented in the study are included in the article/**Supplementary Material**. Further inquiries can be directed to the corresponding authors.
