# Supplementary material for: Recent trends in research on the role of cholesterol in leukemia: a bibliometric and visualization study
Source: Front Immunol. 2025 Jan 23;16:1511827. doi: 10.3389/fimmu.2025.1511827 (PMC11799240; doi:10.3389/fimmu.2025.1511827)
Supplement: Supplementary file 1 [file Table1.docx]

The search formula is ((((((((((((((((((((((((((((((((((((((((((((((((((((((((((((((((((((((((((((((((((((((TS=(Leukemia)) OR TS=(Leukemias)) OR TS=(Leucocythaemia)) OR TS=(Leucocythaemias)) OR TS=(Leucocythemia)) OR TS=(Leucocythemias)) OR TS=("Lymphoblastic Leukemia")) OR TS=("Lymphocytic Leukemia, Acute")) OR TS=("Acute Lymphocytic Leukemia")) OR TS=("Leukemia, Acute Lymphocytic")) OR TS=("Leukemia, Lymphocytic, Acute")) OR TS=(" Leukemia, Lymphoblastic, Acute")) OR TS=("Lymphoblastic Leukemia, Acute")) OR TS=("Lymphoblastic Lymphoma")) OR TS=("Lymphoma, Lymphoblastic")) OR TS=("Acute Lymphoid Leukemia")) OR TS=("Leukemia, Acute Lymphoid")) OR TS=("Lymphoid Leukemia, Acute")) OR TS=("Leukemia, Lymphocytic, Acute, L1")) OR TS=("ALL, Childhood")) OR TS=("Childhood ALL")) OR TS=("Leukemia, Lymphoblastic, Acute, L1")) OR TS=("L1 Lymphocytic Leukemia")) OR TS=("Lymphocytic Leukemia, L2")) OR TS=("Leukemia, Lymphoblastic, Acute, L2")) OR TS=("L2 Lymphocytic Leukemia")) OR TS=("Leukemia, Myeloid, Acute")) OR TS=("Leukemia, Acute Myeloid")) OR TS=("Acute Myelogenous Leukemia")) OR TS=("Acute Myelogenous Leukemias")) OR TS=("Leukemias, Acute Myelogenous")) OR TS=("Leukemia, Acute Myelogenous")) OR TS=("Myelocytic Leukemia, Acute")) OR TS=("Acute Myelocytic Leukemia")) OR TS=("Myelocytic Leukemias, Acute")) OR TS=("Acute Myeloblastic Leukemia")) OR TS=("Acute Myeloblastic Leukemias")) OR TS=("Myeloblastic Leukemias, Acute")) OR TS=("Acute Myeloid Leukemia")) OR TS=("Leukemias, Acute Myeloid")) OR TS=("Myeloid Leukemias, Acute")) OR TS=("Myeloid Leukemia, Acute")) OR TS=("Myeloid Leukemia, Acute, M1")) OR TS=("Acute Myeloid Leukemia without Maturation")) OR TS=("Leukemia, Myeloid, Acute, M2")) OR TS=("Myeloid Leukemia, Acute, M2")) OR TS=("Acute Nonlymphoblastic Leukemia")) OR TS=("Leukemia, Lymphocytic, Chronic, B-Cell")) OR TS=("Small-Cell Lymphoma")) OR TS=("Lymphoma, Small-Cell")) OR TS=("B-Lymphocytic Leukemia, Chronic")) OR TS=("Chronic B-Lymphocytic Leukemia")) OR TS=("Leukemia, Chronic B-Lymphocytic")) OR TS=("B-Cell Leukemia, Chronic")) OR TS=("Chronic B-Cell Leukemia")) OR TS=("Lymphocytic Leukemia, Chronic, B Cell")) OR TS=("Chronic Lymphocytic Leukemias")) OR TS=("Chronic Lymphoblastic Leukemia")) OR TS=("Diffuse Well-Differentiated Lymphocytic Lymphoma")) OR TS=("Lymphocytic Lymphoma, Diffuse, Well Differentiated")) OR TS=("Lymphocytic Lymphoma, Well Differentiated")) OR TS=("Well-Differentiated Lymphocytic Lymphoma")) OR TS=("Lymphoma, Lymphoplasmacytoid, CLL")) OR TS=("CLL Lymphoplasmacytoid Lymphoma")) OR TS=("Small Lymphocytic Lymphoma")) OR TS=("B-Cell Chronic Lymphocytic Leukemia")) OR TS=("B-Cell Malignancy, Low-Grade")) OR TS=("Disrupted In B-Cell Malignancy")) OR TS=("Chronic Lymphatic Leukemia")) OR TS=("Lymphatic Leukemia, Chronic")) OR TS=("Leukemia, Myelogenous, Chronic")) OR TS=("Chronic Myelogenous Leukemia")) OR TS=("Myeloid Leukemia, Philadelphia-Positive")) OR TS=("Myeloid Leukemia, Philadelphia Positive")) OR TS=("Philadelphia-Positive Myeloid Leukemia")) OR TS=("Leukemia, Myelogenous, Ph1 Positive")) OR TS=("Myelogenous Leukemia, Ph1 Positive")) OR TS=("Leukemia, Myeloid, Ph1 Positive")) OR TS=("Myeloid Leukemia, Ph1 Positive")) OR TS=("Granulocytic Leukemia, Chronic")) OR TS=("Leukemia, Chronic Granulocytic")) OR TS=("Myeloid Leukemia, Chronic")) OR TS=("Leukemia, Myeloid, Chronic")) OR TS=("Chronic Myelocytic Leukemia")) OR TS=("Leukemia, Chronic Myelocytic")) OR TS=("Myelocytic Leukemias, Chronic")) OR TS=("Leukemia, Chronic Myeloid") AND (TS=(Cholesterol) OR TS=(Epicholesterol)). The H-index, impact factor (IF), and Journal Citation Reports (JCR) divisions of the journals were acquired from Web of Science (WOS) in a scholarly manner.
